# Biocontrol of chocolate spot disease (*Botrytis cinerea*) in faba bean using endophytic actinomycetes *Streptomyces*: a field study to compare application techniques

**DOI:** 10.7717/peerj.8582

**Published:** 2020-03-09

**Authors:** Sahar A. El-Shatoury, Fuad Ameen, Heba Moussa, Omar Abdul Wahid, Ahmed Dewedar, Saleh AlNadhari

**Affiliations:** 1 Botany Department, Faculty of Sciences, Suez Canal University, Ismailia, Egypt; 2 Department of Botany & Microbiology, College of Science, King Saud University, Riyadh, Saudi Arabia; 3 Department of Marine Biology, Al-Hodeidah University, Al-Hodeidah, Yemen; 4 Department of Plant Protection, College of Agriculture, King Saud University, Riyadh, Saudi Arabia; 5 Department of Plant Protection, College of Agriculture & Veterinary Medicine Farms, Yemen, Ibb University, Ibb, Yemen

**Keywords:** *Vicia fabae*, Pathogenic fungi, Biological control, Sustainable agriculture, Growth promotion agent, Endophytic streptomyces

## Abstract

Sustainable agriculture is needing economic applications for disease control. One possibility is offered by local medical plants. Endophytes of medical plants, such as actinomycetes *Streptomyces* sp. have previously shown antagonistic activities against fungal phytopathogens. In the present field experiment, we aimed to verify the efficiency of endophytic S*treptomyces* against one of the common pathogens, *Botrytis cinerea*, causing chocolate spot disease for faba bean (*Vicia fabae* L.). We tested two strains of *Streptomyces* (MG788011, MG788012) and three techniques to apply the biocontrol agent: (1) coating the seeds with spores, (2) spraying mycelia and (3) spraying the crude metabolites over the plants. The technique using the crude metabolites was the most efficient to prevent the disease symptoms. Both of the endophytic strains diminished the disease symptoms and improved the plant growth. The study offers a potential biological control technique to prevent chocolate spot disease and, at the same time, increase the yields of faba bean in sustainable agriculture.

## Introduction

Sustainable agriculture is looking for novel biocontrol agents and economic techniques to produce and apply the agents. One of the most important protein-rich food legumes worldwide is faba bean (*Vicia fabae* L.) (Stat, 2012). Faba bean is not only food and feed, it offers solutions to sustainable agriculture through its ability to fix atmospheric nitrogen, improve soil fertility, and increase soil carbon storage (Sahile et al., 2008; Jensen, Peoples & Hauggaard-Nielsen, 2010). This strategic crop is unfortunately suffering from many destructive diseases; it is attacked by more than a hundred pathogens, particularly in the wet regions such as the Mediterranean (Karkanis et al., 2018). One of its worldwide diseases, namely chocolate spot disease, brings about losses ranging from a minor to complete failure of the crop. The disease is caused by two fungal species *Botrytis fabae and B. cinerea*.

In the controlling of the chocolate spot disease, certain micronutrient fertilizations as well as chemical fungicides have shown their efficiency (Abeer, Mohd Amin & Martin, 2014). However, chemical fungicides have various ecological side-effects, and they can be found as residues in the crop (Menzel, Gomez & Smith, 2016). Therefore, an emerging area of research is the biological control of the disease. Many bacterial and fungal isolates have shown their antagonistic potential against *Botrytis* infection on different crop plants (Stoddard et al., 2010; Sahile et al., 2011; Haidar et al., 2016; Mbazia, Omri Ben Youssef & Kharrat, 2016; Wang et al., 2018a). However, most studies have been carried out in laboratory conditions. In the field, the biological control treatments have been shown to be less efficient than commercial fungicides (Ramadan, 2014). The efficiency of biocontrol treatments, which were based on the fungi *Trichoderma harzianum* and *Ampelomyces quisqualis*, and the bacteria *Bacillus subtilis*, was observed in one of the rare field experiments (El-Banoby et al., 2013). Therefore, sustainable agriculture is still looking for agents and techniques to effectively control the chocolate spot disease in practice (Stoddard et al., 2010). One group of microorganisms is endophytic actinomycetes, many species of which have shown their potential as biocontrol agents (Misk & Franco, 2011). However, field experiments are lacking.

In selecting proper species as biocontrol agents, attention should be paid not only on the disease inhibition but on the growth promotion of the crop plant. Endophytic actinomycetes are known to accelerate seedling emergence and promote plant establishment and growth directly by fixing atmospheric nitrogen, solubilizing phosphate and by producing phytohormones (Misk & Franco, 2011; Haidar et al., 2016; Dias et al., 2017; Sadeghi et al., 2017; Purushotham et al., 2018; Wang et al., 2018b). Therefore, endophytic actinomycetes are promising organisms to be studied when developing biocontrol agents.

In our previous investigation (Moussa et al., 2011), several endophytic actinomycetes isolated from wild Egyptian medicinal plants showed antagonistic activities against *B. cinerea* when studied *in vitro*. In the present field experiment, we took a step forwards to develop a biocontrol agent against chocolate spot disease and studied, in situ, the following: (a) the efficiency of two *Streptomyces* species as a biocontrol against *B. cinerea* and as a growth promoting agent of faba bean, and (b) different techniques to produce and apply the agent. *Botrytis cinerea* had been chosen originally because it had been more common than *B. fabae* in the study area. This field study will give practical information to develop biological control to prevent chocolate spot disease in faba bean farming.

## Material and Methods

### Experimental design and statistical analyses

The unfertilized sandy farm soil was sown with apparently healthy faba bean seeds (*Vicia fabae* cv. Giza 3). Five seeds were sown per each plot at approximately 30 cm intervals. All seeds germinated and five healthy plants grew in each plot. The actinomycetes treatments were conducted either immediately before sowing the seeds (Seed treatment) or 45 days after sowing the seeds (Spraying treatments; Myc, Met). After 57 days, the plants were infected with *B. cinerea*. The experiment was followed 120 days from sowing.

A full factorial two-way design with three replicates was applied. The factors were *Streptomyces* strains (St 1 and St 2) and techniques. Techniques had four levels: (1) the seed coating treatment (Seed), (2) the mycelium spray treatment (Myc), (3) the metabolites spray treatment (Met) and (4) the control with no treatment (Zero).

A total of 16 treatment combinations (two strains and four techniques) with three replicates was arranged in the field according to the software Minitab 15.1.0.0 (MINITAB® Minitab Inc.) treatments arrangement scheme. An area of 4 m × 4.5 m was prepared and divided into three blocks, each containing two rows. One row consisted of eight plots, each for different treatment combinations. The effect of the treatments on the disease assessment variables were analyzed with two-way ANOVA followed by Tukey’s test using MINITAB. *P* < 0.05 was considered as significant.

An additional treatment representing healthy plants, where the pathogen *B. cinerea* was not infected, was prepared as triplicate in a solo side-row (Uninfected). The uninfected plants were used to study the effects of the treatments to the growth of the plants. The differences in the plant vigor variables between the treated plants and the healthy uninfected plants were analyzed using *t*-tests.

The area was irrigated by flooding with tap water regularly. The experiment was observed daily to record any changes.

### Endophytic actinomycetes

The endophyte *Streptomyces* sp. were isolated from a white wormwood shrub *Artemisia herba-alba* and identified by chemotaxonomic methods in our previous study (Moussa et al., 2011). For the present study, we first identified the species using DNA techniques. Actinomycetes mycelia were harvested after growing in starch casein broth (SCB) supplemented with 34% sucrose. Mycelia were washed with distilled water and the DNA was extracted using the method described by Kumar, Miao & Wyatt (2010). The 16S rRNA gene was amplified in an automated thermal cycler (Master cycler, Eppendorf, Germany) in 100 µl reactions using the universal primer set 27f and 1492r. The PCR product was purified using EZ-10 Spin Column Plant PCR kit (Bio Basic Inc., Canada) according to the manufacturer’s instructions. Conditions for amplification and sequencing were described previously (Hall, 1999). The sequences were compared with 16S rRNA gene sequences in the National Center for Biotechnology Information (NCBI) database using the BLAST program and the accession numbers obtained were *Streptomyces* MG788011 and *Streptomyces* MG788012.

### Treatments

The treatments were prepared for both *Streptomyces* strains as follows. First, spore suspensions were prepared. The isolate was first grown on starch casein agar (SCA) at 27 °C for 5 days until a complete sporulation had occurred. Three milliliters of sterilized distilled water plus one drop of 0.1% Tween 80 (wetting agent) were added to a plate, scraped gently with a loop to loosen the spores and the mycelium. A loopful of the mycelia and spores was inoculated into a 250 ml of starch casein broth (SCB) in 500-ml Erlenmeyer flask and shaken on a rotator shaker 100 rpm min^−1^ at 27 °C for 7–10 days. In the experiments, the distilled water suspensions were adjusted to 10^7^ CFU ml^−1^ using first hemocytometer and thereafter verified by plating on SCA for five days.

The first technique, the seed treatment (Seed) was prepared as follows. The disinfected faba bean seeds were soaked (24 h) in the spore suspension (explained above). The zero treatment seeds were soaked in distilled water. Seeds were dried in a fume hood for 24 h. The seeds were sown to the experimental sites.

The second technique, the mycelium spray treatment (Myc) was prepared as follows. From the spore suspension, the cells were harvested by centrifugation (3,000 rpm) for 7 min. The resultant pellet was homogenized and suspended in 10 ml sterile distilled water. Mycelia were washed three successive times, and harvested by centrifugation, The CFU concentration was adjusted as explained above. An aliquot of 5 ml of the suspension was sprayed over each plant. Zero treatment was sprayed with tap water.

The third technique, the metabolites spray treatment (Met) was prepared as follows. Crude metabolites were obtained from the SCB broth culture (10 days old) by centrifuging at 3,000 rpm for 7 min. Metabolites as such was sprayed over the seedlings (5 ml per seedling). In both spraying treatments, a plastic sprayer, a plastic tent, and a plastic cover over the soil were used to control the spraying and to prevent the unwanted spreading of the treatment agents.

### *Botrytis cinerea* infection

*Botrytis cinerea* had been isolated from artificially infected bean leaves as described in Moussa et al. (2011). For the present experiment, *B. cinerea* was grown on PDA and incubated at 18 °C for 7–10 days until a complete sporulation had occurred. Three millimeters of sterilized distilled water plus one drop of 0.1% Tween 80 solution were added to the plates before they were scraped gently with a loop to loosen the spores and the mycelium. The harvested inoculum was homogenized in a glass mortar and a distilled water suspension was adjusted to the concentration of approximately 1  × 10^6^ spores/ml using a hemocytometer (the counts were further verified by culturing on PDA plates). An aliquot of 5 ml of the *B. cinerea* pathogen suspension was sprayed over each plant. The plants were covered with a plastic cover for 24 h to make sure the infection. Faba bean plants revealing a 100% infection with *B. cinerea* infection was confirmed by typical chocolate spot disease symptoms appearance after two days of the artificial infection.

### Disease assessment variables

Disease assessment and plant vigor variables were observed daily, 70–120 days from sowing. The final disease assessments were done 120 days after the sowing. For disease severity percent (DS %), the symptoms were first assessed using the class rate scale of Bernier et al. (1993): 1 = no disease symptoms or very small specks (highly resistance), 3 = few small discrete lesions (resistant), 5 = some coalesced lesions with some defoliation (moderate resistant), 7 = large coalesced sporulating lesions, 50% defoliation and some dead plant (susceptible), 9 = extensive lesions on leaves, stems and pods, severe defoliation, heavy sporulation, stem girdling, blackening and death of more than 80% of plants (highly susceptible). Disease severity percent was calculated according to Hanounik & Maliha (1986):

\begin{eqnarray*}& & \mathrm{DS}\text{%}= \frac{\sum (\mathrm{NPC}\times \mathrm{CR})}{(\mathrm{NIP}\times \mathrm{MSC})} \times 100. \end{eqnarray*}

Where NPC = number of leaflets in each class rate, CR = class rate, NIP = number of infected leaflets, MSC = maximum CR.

Disease incidence percent (DI %) was the percentage of infected plants from all plants. Diseased area percent (DA %) was calculated according to the equation developed by (Shimizu, Yazawa & Ushijima, 2009). For calculating the area of lesions, the leaflets and leaves were photographed using a digital camera (Sony), and images were then painted white using Adobe Photoshop CS3 extended v.10.0 (Adobe systems incorporated) and saved again in TIFF format. Finally, the white areas of the lesions were measured with the public domain program Image J 1.37 (developed by the US National Institutes of health and available at (http://rsb.info.nih.gov/nih-image/). DA% was calculated as the percentage of the lesion area from the total leaf area.

### Plant vigor variables

The faba bean plant growth (plant height, branching, number of leaves) and yield (plant total fresh weight, plant total dry weight, number of pods per plant) were measured after 120 days of sowing. Two plants from each plot from the three blocks (six plants per treatment) were randomly selected.

## Results

The uninfected plants were healthy with no disease symptoms. In general, both *Streptomyces* strains and techniques had significant effects on the three disease assessment variables, DS %, DI % and DA %. In two-way ANOVA, the *Streptomyces* strains and techniques had a significant interaction for each variable (Table S1). This indicated that the effects of the techniques varied depending on the combination of the treatments.

**Figure 1:**
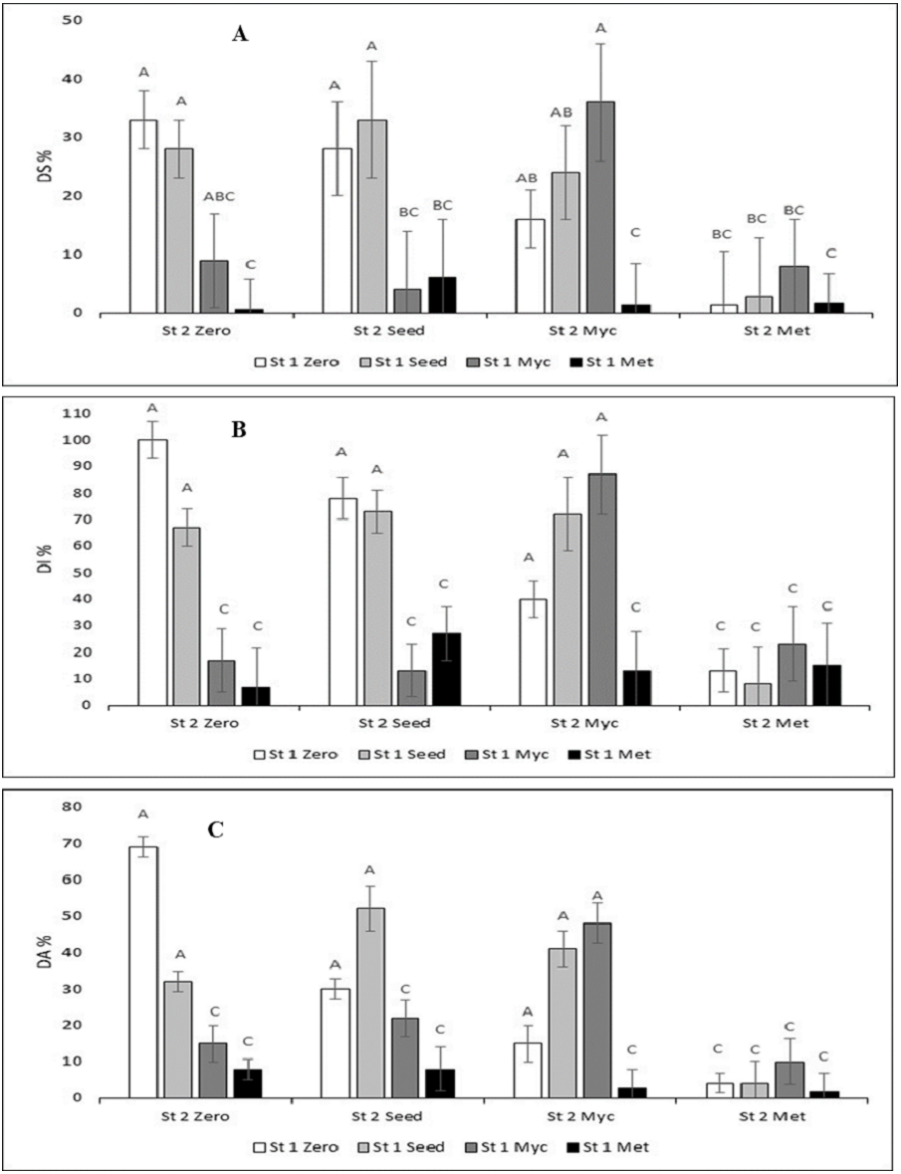
Disease assessment variables (DS%, disease severity; DI%, disease incidence; DA%, diseased area) in the combinations of the treatments with the *Streptomyces* strains and the techniques to apply the biocontrol agent against *Botrytis cinerea* infection. Some of the error bars (SD, *n* = 3) are cut by the scale of the *x*-axis. Means with the same letters over the columns were not statistically significant (Tukey’s *p* < 0.05).

Due to significant interactions, Tukey’s tests were performed separately for the different *Streptomyces* stains. The multiple comparisons (Tukey, *p* < 0.05) between the different treatment combinations revealed in general that DI % and DA % formed identical groups of the treatments (Fig. 1). DS % grouping was slightly different (Fig. 1). Based on these variables, the most effective technique that decreased disease symptoms was the metabolites spray treatment (Met) of both St 1 and St 2. DS % had some more variation, yet the group of combinations where the infected control belonged to (Fig. 1) was about the same for all variables, and the Seed treatments appeared to be the least efficient. The seed treatments did not differ significantly from the control for any of the variables.

DS % was 33% ± 5 for the Zero treatment (Infected Control) (Fig. 1A; the first white column; no *Streptomyces* treatment). DS% was lowest when either St 1 Met (ca. 1–6%; black columns) or St 2 Met (ca. 1–8%, column group at right) was added. The lowest DS % of all was for the St 1 Met + St 2 Zero treatment, 0.7 ± 2% (right black column). Thereafter, DS % increased in the order: St 1 Zero + St 2 Met (1.5 ± 9%) = St 1 Met + St 2 Met (1.5 ± 4%) <St 1 Met + St 2 Myc (1.7 ± 4%) <St 1 Seed + St 2 Met (2.8 ± 10%) <St 1 Myc + St 2 Seed (4.0 ± 10%). For the rest of the treatments, DS% was up to 36 ± 10%.

DI% and DA% graphs showed much similar results to DS%. DI was 100 ± 7% for Zero (Fig. 1B middle graph). The lowest DI was St1-Met was 6.6 ± 7.1%. DA was 69 ± 3% for Zero (Fig. 1C). The lowest DA% was for St 1Met + St 2 Met, it was 2 ± 5%.

Plant growth and yield varied a lot, generally all *Streptomyces* treatments had at least slightly higher values compared to uninfected healthy plants that were water sprayed. However, the most efficient treatments were the ones where Met (either St 1 or St 2) was included. Met treatments differed significantly (*t*-test, *p* < 0.05) from the uninfected control regarding most plant vigor variables (Table 1). Uninfected plants’ shoot length was 86.0 ± 3.2 cm. For the St 1 Met treatment it was 99.0 ± 0.8 cm, and for St 2 Met it was 111.6 ± 0.5 cm. The respective comparison for the plant dry weight is 10.5 ± 0.2 g, 12.3 ± 0.8 g and 18.9 ± 1.2 g.

**Table 1:** Plant vigor (growth and yield) variables (mean ± SD, *n*= 3) in different selected biocontrol treatments of *Botyris cinerea* infected faba bean plants in comparison to uninfected (water sprayed) plants (in bold). St 1 and St 2 refer to the two *Streptomyces* strains used to extract mycelia (Myc) or metabolites (Met). Seed refer to the seed treatment with St 1 or St 2 spore suspension.

Treatment			
	**Plant growth**		
	**Shoot length (cm)**	**Root length (cm)**	**Leaves No.**
St 1 Met	99.0 ± 0.8^*^	11.5 ± 0.8	18.0 ± 0.8^*^
St 2 Met	111.6 ± 0.5^*^	12.2 ± 0.4^*^	19.4 ± 0.5^*^
St 1 Met + St 2 Met	91.5 ± 4.2	12.5 ± 0.9^*^	20.7 ± 1.6^*^
St 1 Met + St 2 Myc	92.2 ± 4.6	12.3 ± 0.8^*^	18.7 ± 1.8^*^
St 1 Seed + St 2 Met	91.2 ± 3.9	12.0 ± 1.0^*^	17.6 ± 1.9
St 1 Myc + St 2 Seed	85.3 ± 4.0	11.3 ± 1.1	18.3 ± 1.4^*^
**Uninfected plants**	**86.0 ± 3.2**	**10.2 ± 0.5**	**15.4 ± 0.5**
	**Plant Yield**		
	**Fresh Weight (g)**	**Dry Weight (g)**	**Pod No.**
St 1 Met	42.8 ± 0.8	12.3 ± 0.8^*^	2.4 ± 0.8^*^
St 2 Met	114.5 ± 10.3^*^	18.9 ± 1.2^*^	3.5 ± 0.8^*^
St 1 Met + St 2 Met	85.2 ± 14.0^*^	17.6 ± 1.7^*^	3.5 ± 0.7^*^
St 1 Met + St 2 Myc	85.2 ± 14.4^*^	21.6 ± 1.5^*^	3.8 ± 0.7^*^
St 1 Seed + St 2 Met	59.6 ± 4.1^*^	14.8 ± 1.9^*^	3.5 ± 0.7^*^
St 1 Myc + St 2 Seed	56.6 ± 14.1	13.4 ± 1.7^*^	3.0 ± 0.7^*^
**Uninfected plants**	**45.5 ± 1.2**	**10.5 ± 0.2**	1.5 **± 0.3**

**Notes.**

* Significant difference compared to uninfected plants (*t*-test, *p* < 0.05).

## Discussion

In developing biocontrol agents to treat plant fungal pathogenic diseases, many different organisms could be utilized. Various actinomycetes species are known to have antagonistic activities against many pathogenic microorganisms (Moussa et al., 2011; Ahmad et al., 2017; Martinez-Klimova, Rodriguez-Pena & Sánchez, 2017; Sadeghi et al., 2017; Fabrizius et al., 2018). Many actinomycetes can produce antifungal or antibacterial compounds or out-compete the pathogens (El-Tarabily & Sivasithamparam, 2006; Gomes, Duarte & De Freire Bastos do, 2017; Lee et al., 2018). The genus *Streptomyces* belonging to phylum Actinobacteria is well known for its antimicrobial properties. Various *Streptomyces* species, isolated from soils, as well as, from many medicinal plants, have been observed to have antagonistic effects against various pathogens (Cao et al., 2005; Prapagdee, Kuekulvong & Mongkolsuk, 2008; Mingma et al., 2014; Himaman et al., 2016; Swarnalakshmi, Senthilkumar & Ramakrishnan, 2016). *Streptomyces* efficiency against *Botrytis* infection has been observed several times (Bi & Yu, 2016; Duraipandiyan, Al-Dhabi & Ignacimuthu, 2016; Salla, Astarita & Santarém, 2016; Boukaew, Prasertsan & Sattayasamitsathit, 2017; Lian et al., 2017; Lyu et al., 2017). Our literature search gave us a lot of knowledge about the possibly efficient biological agents, mostly studied *in vitro*, to control chocolate spot disease. However, field studies giving information are scarce. The challenge in this research field is to develop the practical applications that could be utilized in sustainable agriculture, taking the local conditions and safe-application of microorganisms into account. Therefore, we ended up to choose local medicinal plants and their endophytic actinomycetes, and produce potential biocontrol agents from them. We also focused on the practical application and carried out a field experiment where we observed the biocontrol potential of endophytic *Streptomyces*. We suggest that endophytic *Streptomyces* treatment is safe and efficient against *B. cinerea* infection causing chocolate spot disease on faba bean. However, more development is needed.

In our field experiment, we tested different techniques to produce and apply the biocontrol agent. The crude metabolites from *Streptomyces* SCB broth culture appeared to be the most effective. The chocolate spot disease symptoms were clearly diminished after spraying the metabolites over *B. cinerea* infected faba bean plants. The combination of mycelia from both endophytic *Streptomyces* strains appeared to be of less efficiency.

Although the seed treatments did not differ significantly from the control, it seemed that they reduced the disease symptoms to some extent. This may be an indirect systemic effect. *Streptomyces* are known for their many beneficial roles in the roots of many plants, as reviewed recently by (Franco-Correa & Chavarro-Anzola, 2016). Indirect mechanisms inducing systemic resistance against phytopathogens have been observed. *Streptomyces* sp., *in planta*, seem to improve plant defense mechanisms, for instance, through promoting its growth (Schrey & Tarkka, 2008). Root inoculation with *Streptomyces* sp. have been observed to reduce, for instance, the needle infection of *B. cinerea* in Norway spruce as well as the powdery mildew infection of the leaves of pedunculate oak (Lehr et al., 2008; Kurth et al., 2014). This aspect in faba bean disease control deserves further research.

Knowledge on biocontrol agents is needed worldwide and locally. It is easier to develop applications for a narrower than a wide range of conditions (Haidar et al., 2016). Local solutions using local plants offer economic and sustainable possibilities for sustainable agriculture, especially in less-developed countries. We isolated endophytic actinomycete *Streptomyces* from a local shrub *Artemisia herba-alba*, commonly known as desert or white wormwood. The plant grows commonly on dry steppes in the Mediterranean region, and has been used against various diseases traditionally.

Our *Streptomyces* treatments had growth-promoting abilities, which we assessed by measuring several plant vigor variables. Although the *Streptomyces* strains did not differ remarkably in their efficiency to inhibit *B. cinerea*, they differed in their growth promoting efficiency to some extent. The first *Streptomyces* strain’s crude metabolite treatment (St 1 Met) caused ca. 15% increase in the shoot length and dry weight compared to uninfected healthy plants. However, the crude metabolites from the second *Streptomyces* strain (St 2 Met) was a more efficient growth promoting agent; the plant dry weight was even 80% higher after the St 2 Met spraying treatment, compared to uninfected healthy plants. As mentioned above, the two species had almost equal ability to diminish the chocolate spot disease symptoms. This shows the complexity of the assessment and the need for detailed studies. Unfortunately, we were not able to test the duration of action of the treatment. A lower duration of action of biocontrol agents compared to chemical agents have been observed elsewhere (Bardin et al., 2015). Therefore, the duration of action of our biocontrol treatment as well as the need to re-treat the plants are still to be studied.

Several other aspects in the further developing of this biocontrol technique must be taken into consideration; a multi-criteria approach is needed (Bouaoud et al., 2018). Several growth inhibition and growth promotion mechanisms are acting at the same time. The optimizing of the procedure needs more work both with the techniques and the bacterial species chosen. The species have different potentials as observed recently. When 72% of the actinobacterial isolates produced siderophores and inhibited *B. cinerea* infection, only 11% of the isolates showed phosphate solubilization ability (Misk & Franco, 2011). At best, biocontrol agents can be even more efficient than commercial fungicides, as was observed for *Botrytis* infection in faba bean when the plant extracts of *Eugenia caryophyllus* was compared to fungicide Ridomil-MZ (Hoda, Migahed & Nofel, 2016). It is not clear if the metabolites as such, or its content of endophytic inocula, would be more effective in controlling the *Botrytis* infection, because the centrifugation we used did not exclude all cells and spores from the metabolite treatment. The next step in our studies is to compare the *Streptomyces* treatments to chemical fungicides and to go deeper into the mechanisms of action in both the spraying and seed treatments.

## Conclusions

The severity of chocolate spot disease was reduced by the endophytic *Streptomyces* treatments carried out in the field experiment. The crude metabolites of both *Streptomyces* strains prevented disease symptoms remarkably when sprayed over the seedlings before the *B. cinerea* infection. The *Streptomyces* metabolites appeared to be an environmentally safe agent, not only to hinder the disease but also to promote the plant growth. The technique has the potential to be developed to control and prevent chocolate spot disease, and at the same time, increase the yields of faba bean in sustainable agriculture.

##  Supplemental Information

10.7717/peerj.8582/supp-1 Table S1 Summary two-way ANOVA tables for disease incidence variables

10.7717/peerj.8582/supp-2 Data S1 Raw Data

## Additional Information and Declarations

### Data Availability

The following information was supplied regarding data availability: Data is available at GenBank: MG788011 and MG788012.
