# Supplementary material for: Biocontrol of chocolate spot disease (Botrytis cinerea) in faba bean using endophytic actinomycetes Streptomyces: a field study to compare application techniques
Source: PeerJ. 2020 Mar 9;8:e8582. doi: 10.7717/peerj.8582 (PMC7067178; doi:10.7717/peerj.8582)
Supplement: Table S1 [file peerj-08-8582-s001.docx]

Table S1. Summary two-way ANOVA tables for disease incidence variables. DS% = disease severity, DI% = disease incidence, DA% = disease area percent.

| Variable | Source | df | F-value | P-value |
| --- | --- | --- | --- | --- |
| DS% | Strains | 1 | 1.02 | 0.327 |
|  | Techniques | 3 | 3.27 | 0.049 |
|  | Interaction | 3 | 4.52 | 0.018 |
|  | Error | 16 |  |  |
|  |  |  |  |  |
| DI% | Strains | 1 | 2.02 | 0.174 |
|  | Techniques | 3 | 5.05 | 0.012 |
|  | Interaction | 3 | 9.56 | 0.001 |
|  | Error | 16 |  |  |
|  |  |  |  |  |
| DA% | Strains | 1 | 1.84 | 0.194 |
|  | Techniques | 3 | 6.86 | 0.003 |
|  | Interaction | 3 | 9.20 | 0.001 |
|  | Error | 16 |  |  |
|  |  |  |  |  |
