# Supplementary material for: Biocontrol of chocolate spot disease (Botrytis cinerea) in faba bean using endophytic actinomycetes Streptomyces: a field study to compare application techniques
Source: PeerJ. 2020 Mar 9;8:e8582. doi: 10.7717/peerj.8582 (PMC7067178; doi:10.7717/peerj.8582)
Supplement: Data S1 [file peerj-08-8582-s002.docx]

**Supplementary File (Row Data)**

**
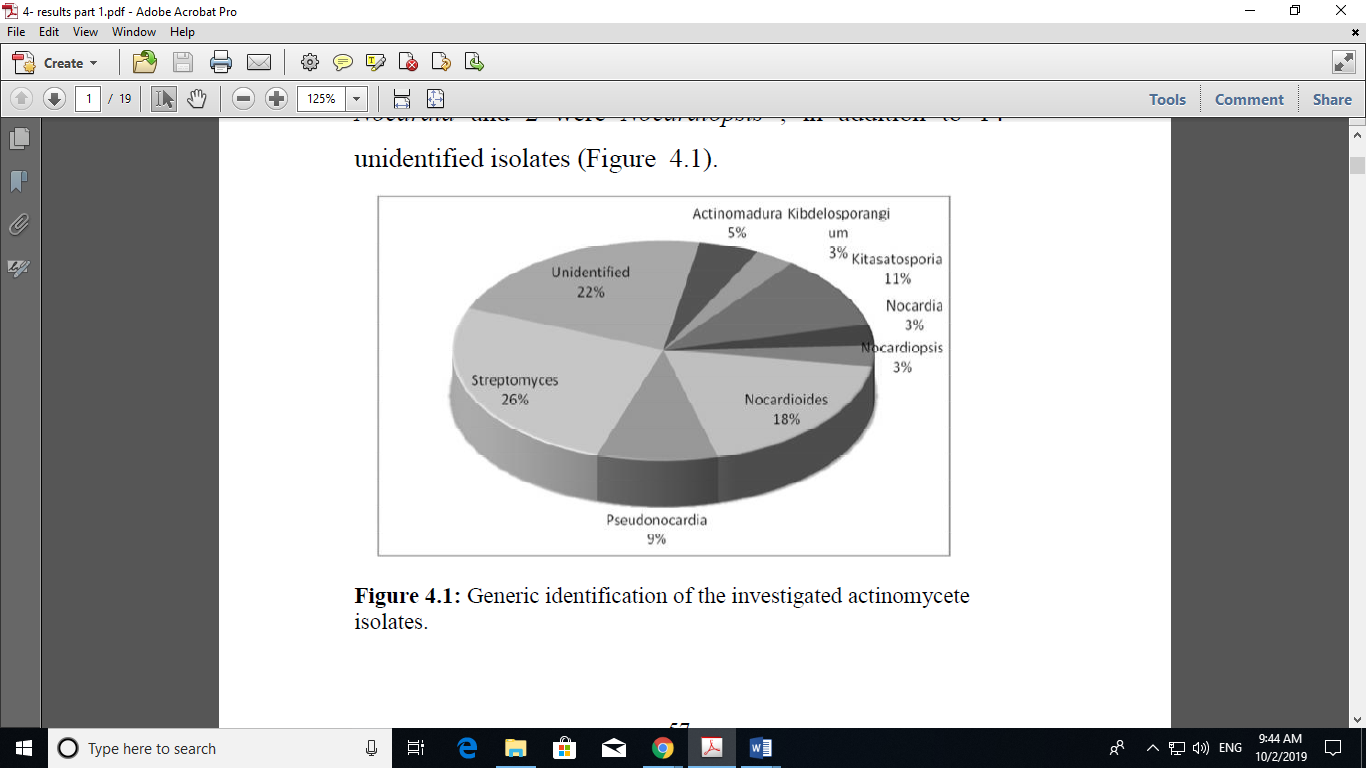
**

Figure 1: Generic identification of the investigated actinomycete isolates


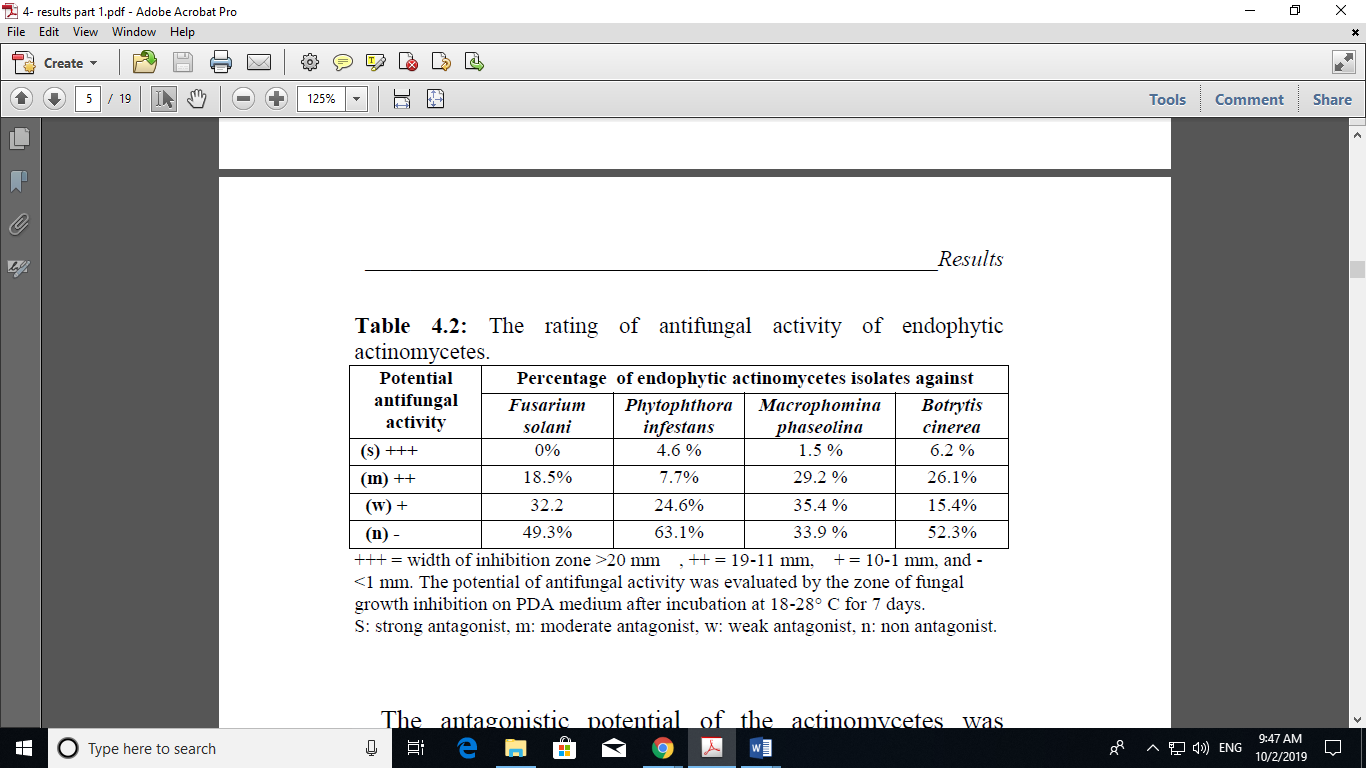
 Table 1: The rating of antifungal activity of endophytic actinomycetes.


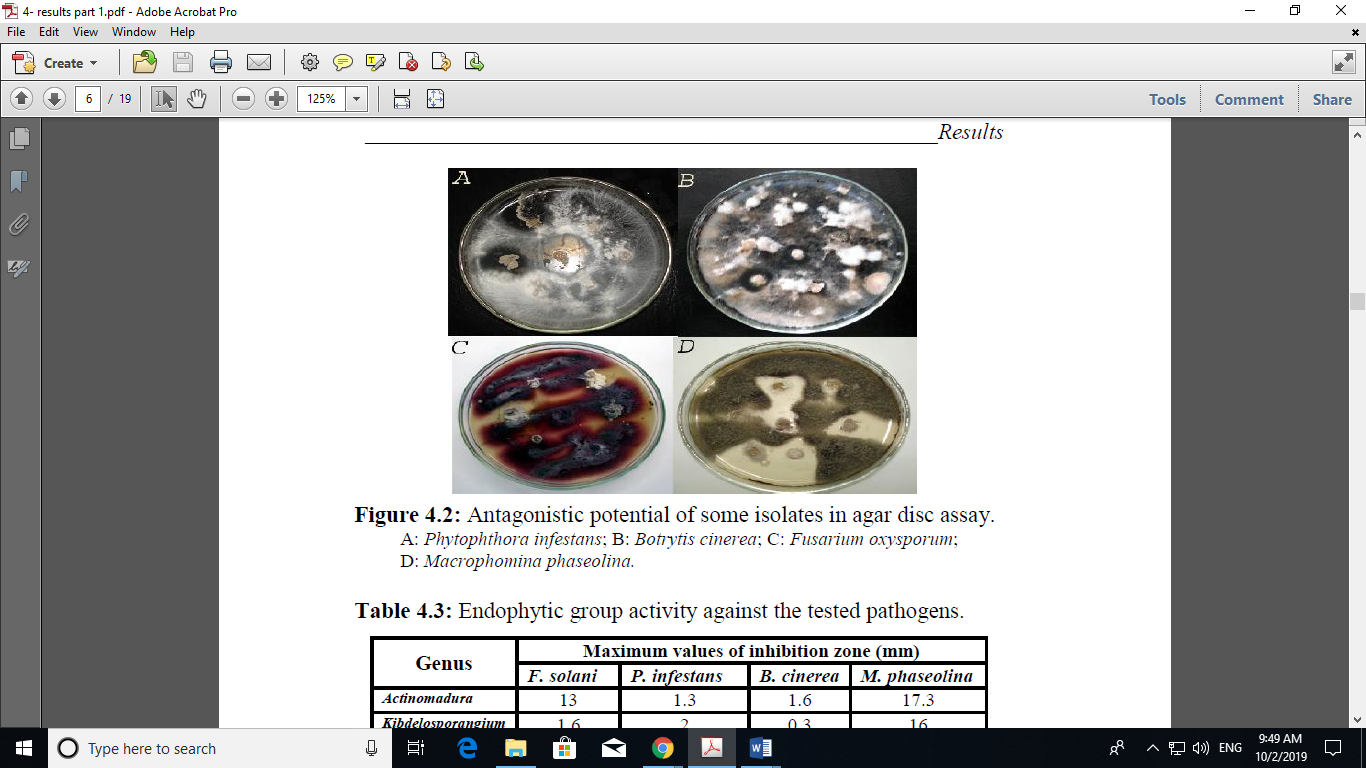


Figure 2: Antagonistic potential of some isolates in agar disc assay. A: *Phytophthora infestans*; B: *Botrytis cinerea*; C: *Fusarium oxysporum*; D: *Macrophomina phaseolina*.


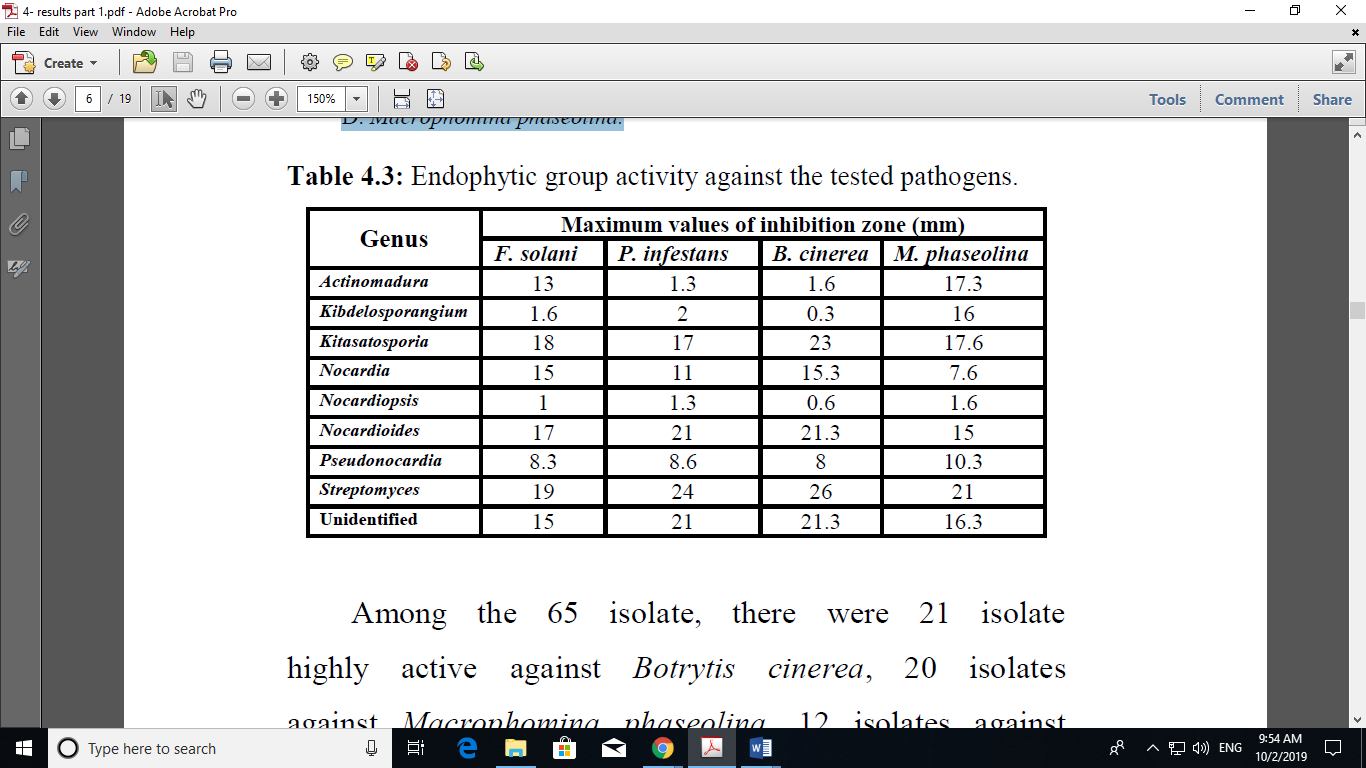
 Table 2: Endophytic group activity against the tested pathogens.


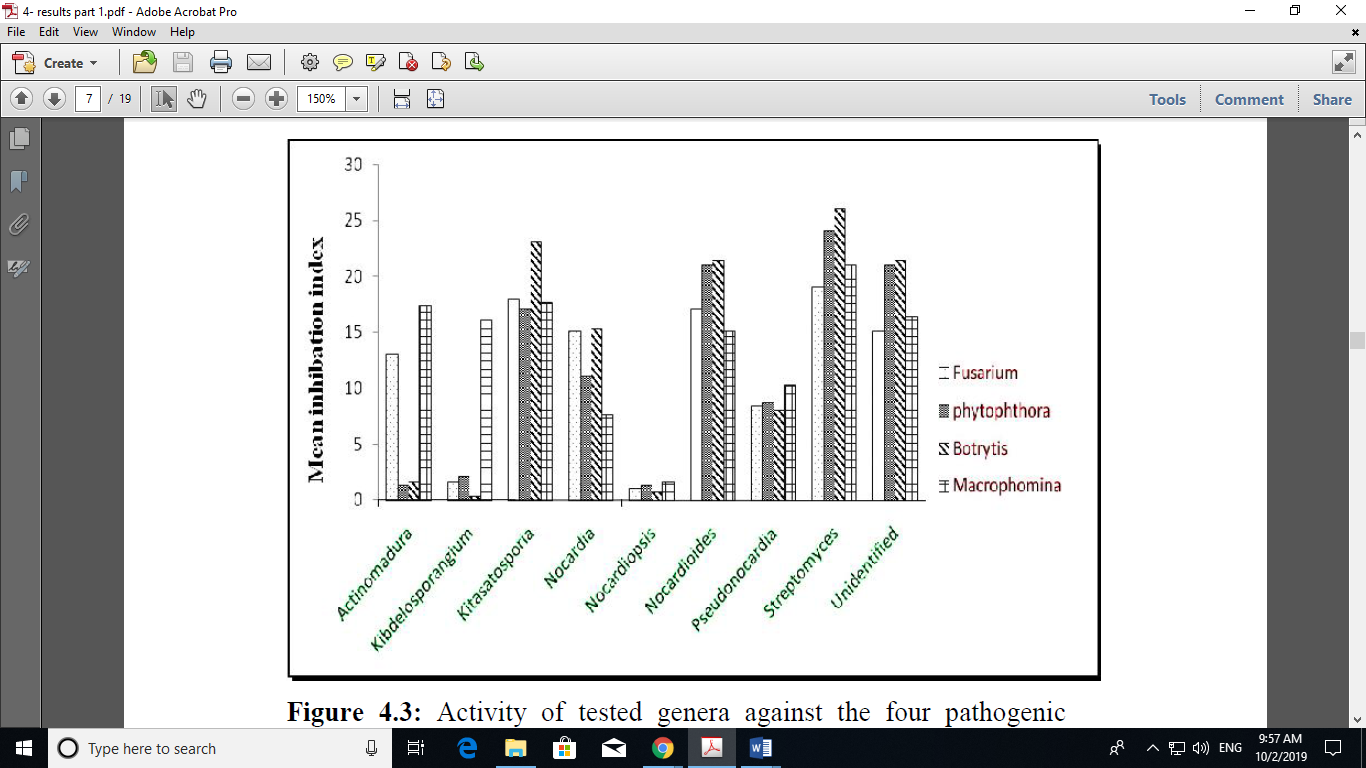


Figure 3: Activity of tested genera against the four pathogenic fungi.


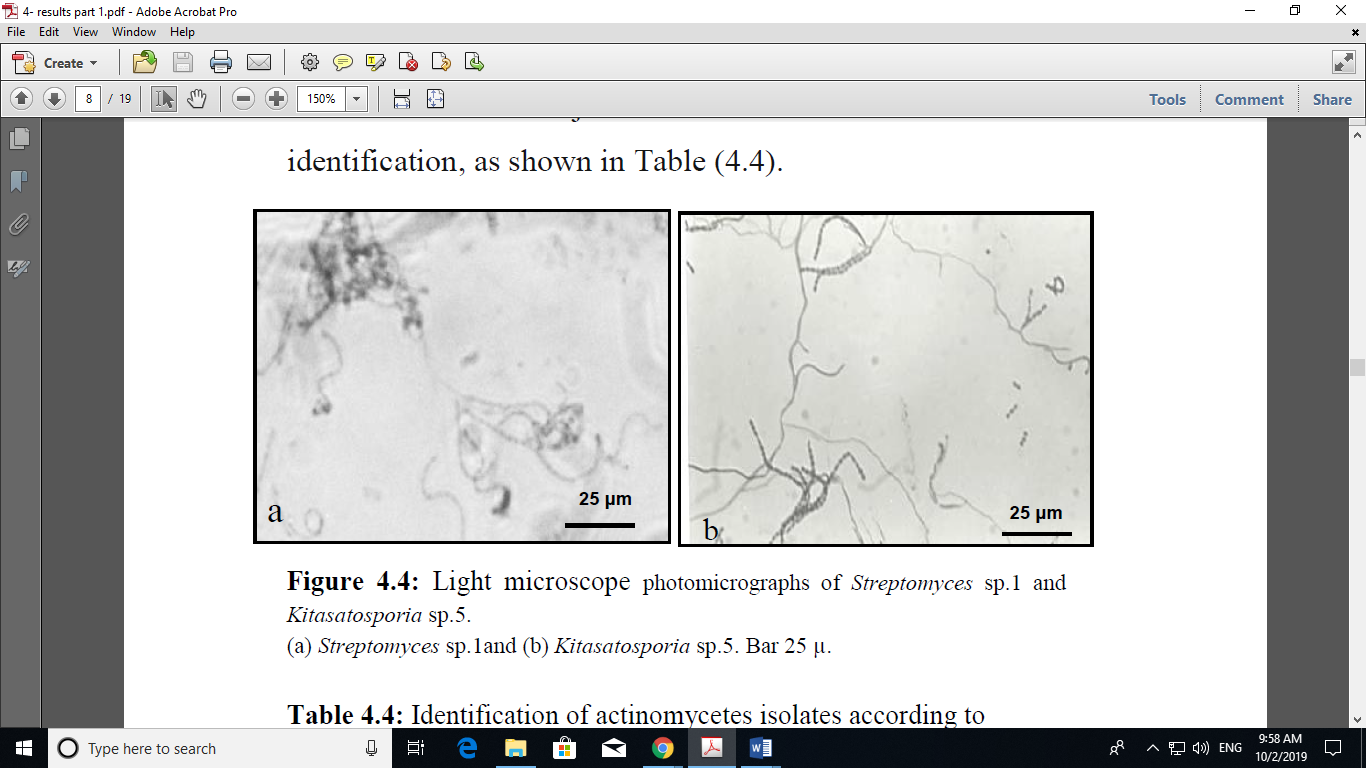


Figure 4: Light microscope photomicrographs of *Streptomyces* sp.1 and *Kitasatosporia* sp.5. (a) *Streptomyces* sp.1and (b) *Kitasatosporia* sp.5. Bar 25 μ.

Table 3: Identification of actinomycetes isolates according to morphological and chemotaxonomic criteria.


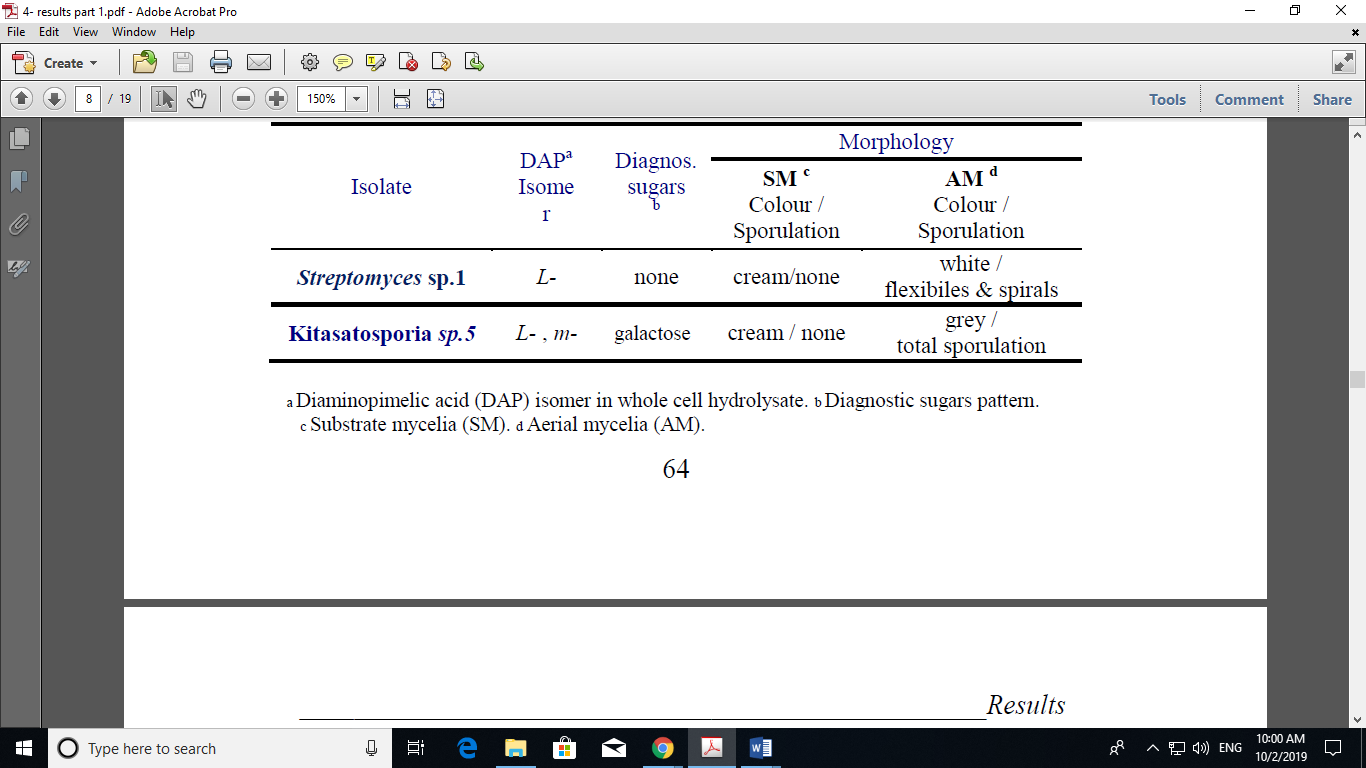


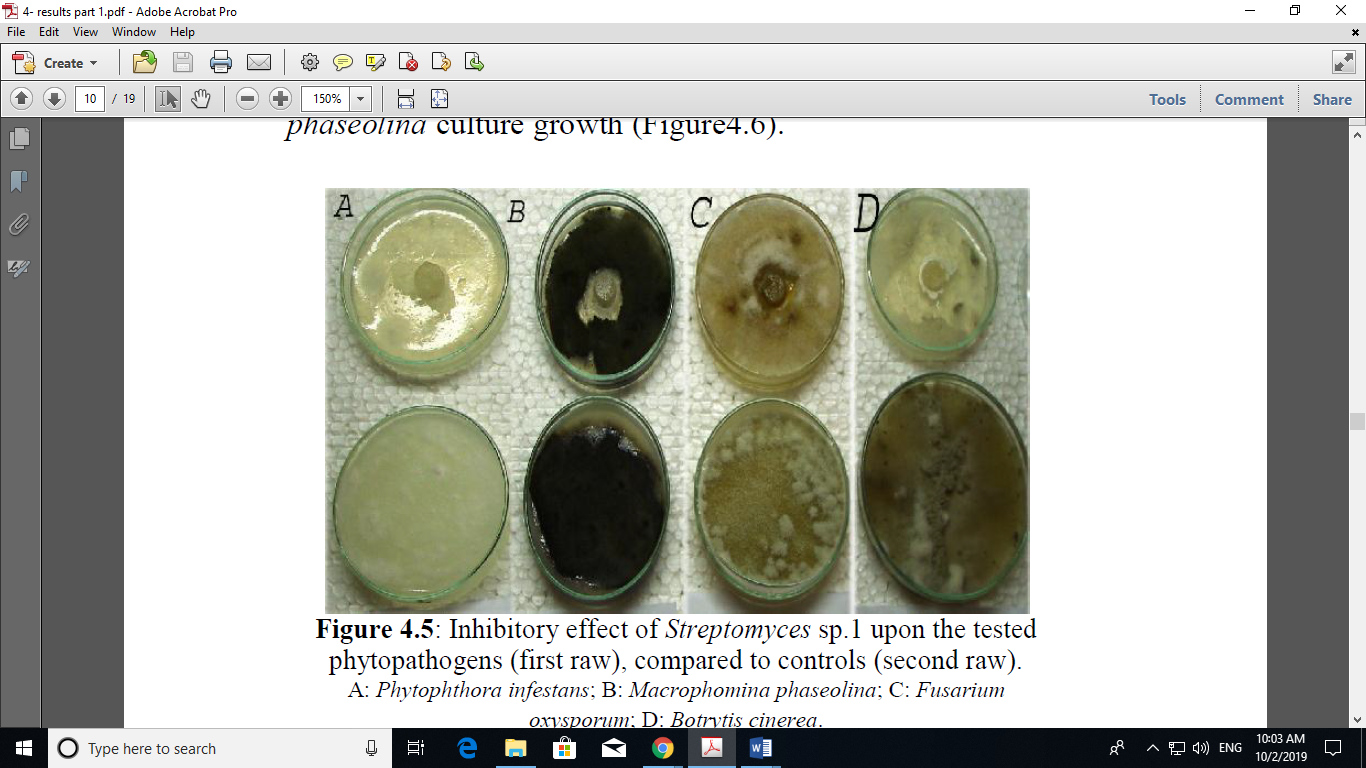


Figure 5: Inhibitory effect of Streptomyces sp.1 upon the tested phytopathogens (first raw), compared to controls (second raw). A: *Phytophthora infestans*; B: *Macrophomina phaseolina*; C: *Fusarium oxysporum*; D: *Botrytis cinerea*.

Figure 6: Inhibitory effect of *Kitasatosporia* sp.5 upon the tested phytopathogens (first raw), compared to controls (second raw). A: *Phytophthora infestans*; B: *Macrophomina phaseolina*; C: *Fusarium oxysporum*; D: *Botrytis cinerea*.

Table 4: Assessment of in vitro antifungal activity of the actinomycetes metabolites, after subculture vitality testing of the phytopathogens.


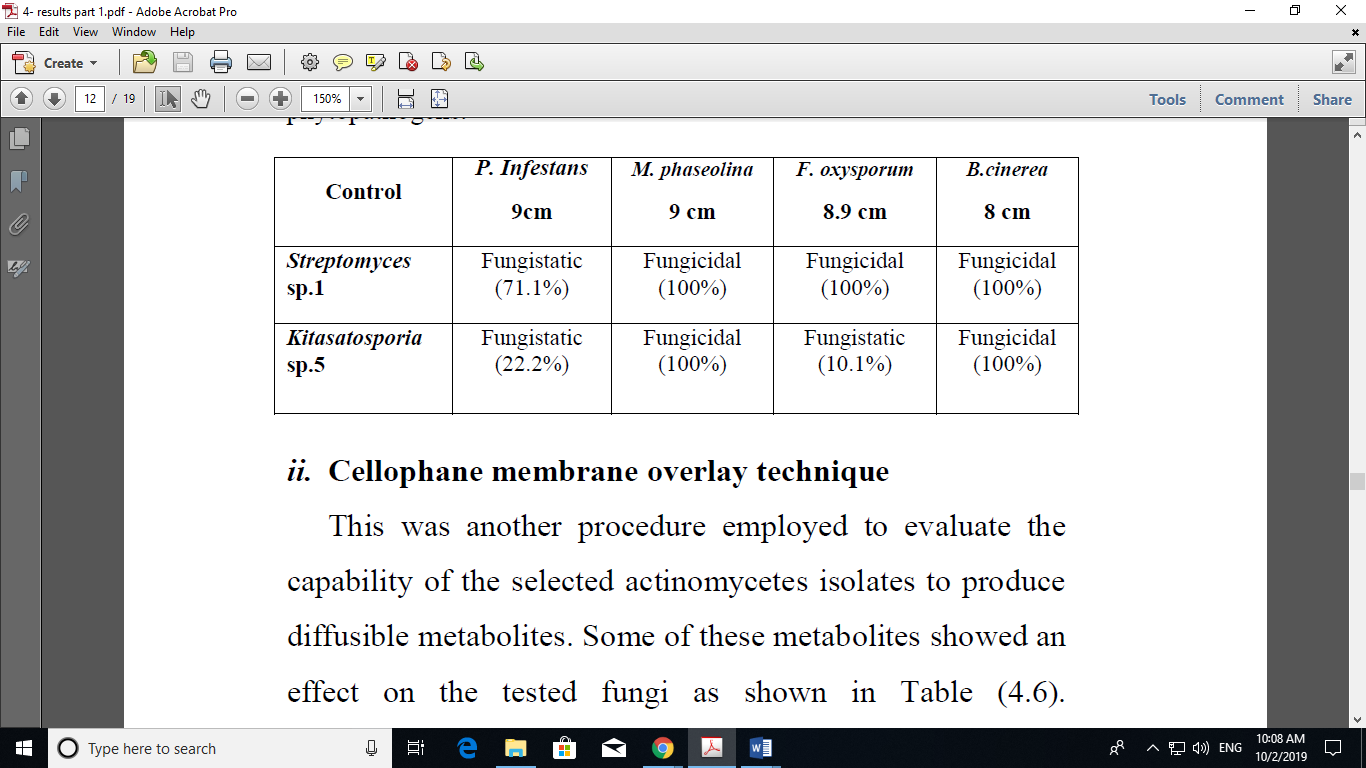


Table 5: Assessment of in vitro antifungal activity of actinomycetes diffusible metabolites on the cellophane membrane.


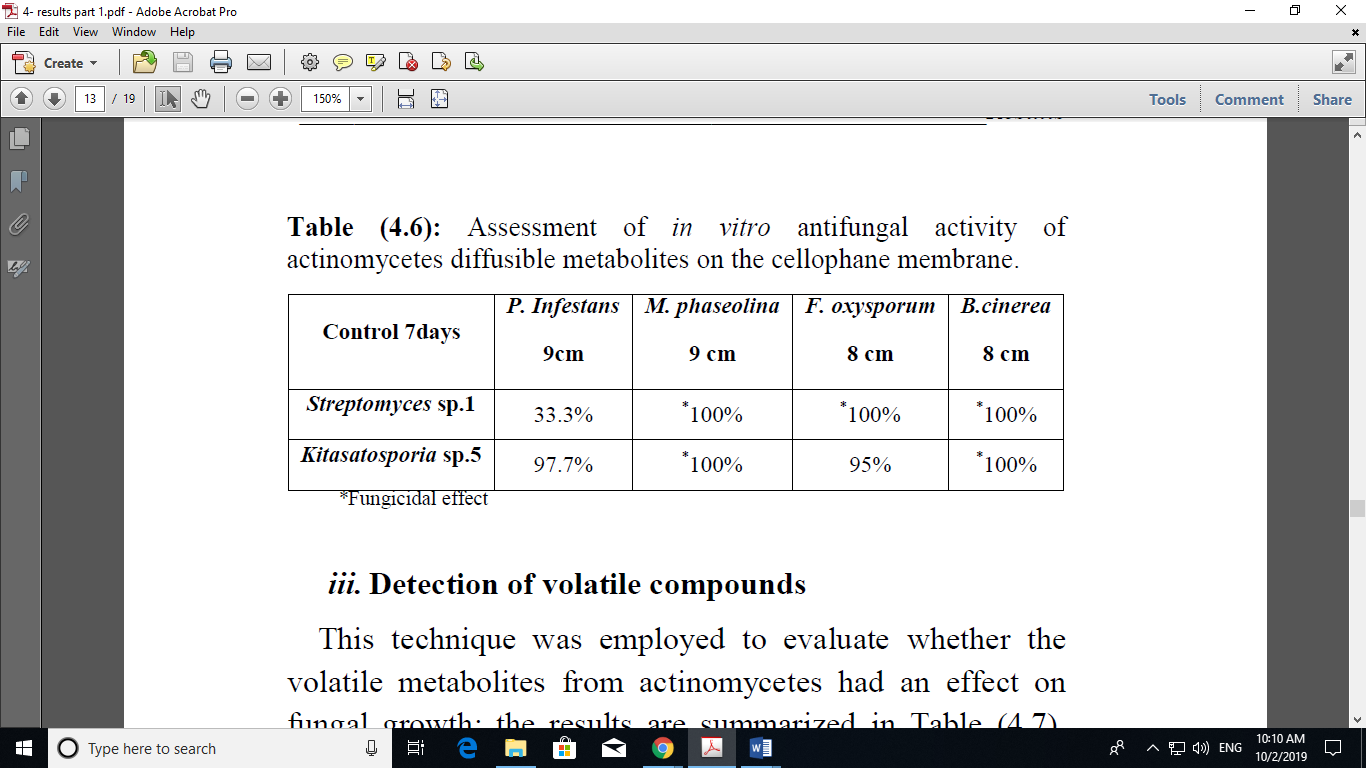


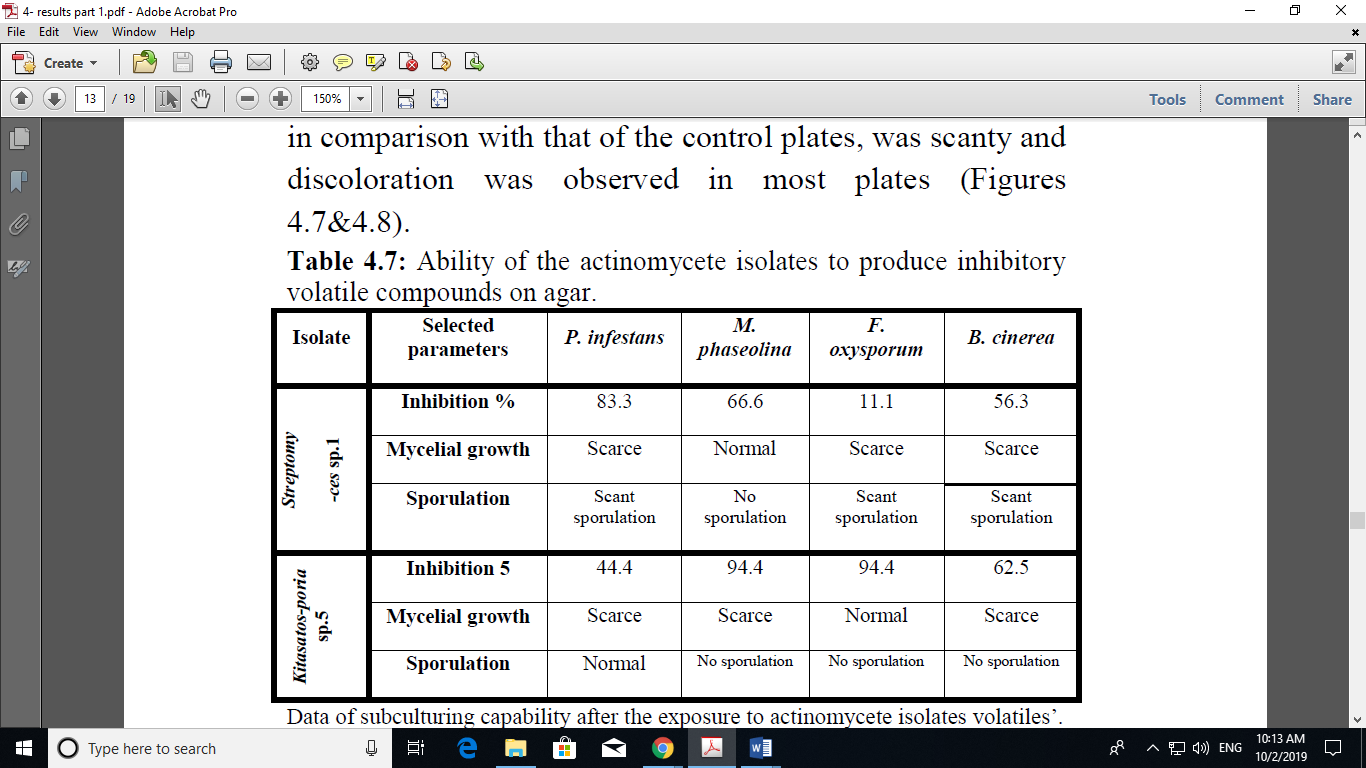
Table 6: Ability of the actinomycete isolates to produce inhibitory volatile compounds on agar.

Figure 7: Inhibitory effects of Streptomyces sp.1volatile compounds on fungal growth. A: P. Infestans; B: *M.* *phaseolina*; C: *F. oxysporum*; D: *B. cinerea*. (1st raw: *Streptomyces* sp.1; 2nd raw: exposed pathogens; 3rd raw: control pathogens).

Figure 8: Effect of volatiles produced by Kitasatosporia sp.5 on the phytopathogens. A: M. phaseolina; B: P. Infestans; C: F. oxysporum; D: B. cinerea. (1st raw: exposed pathogens; 3rd raw: control pathogens).


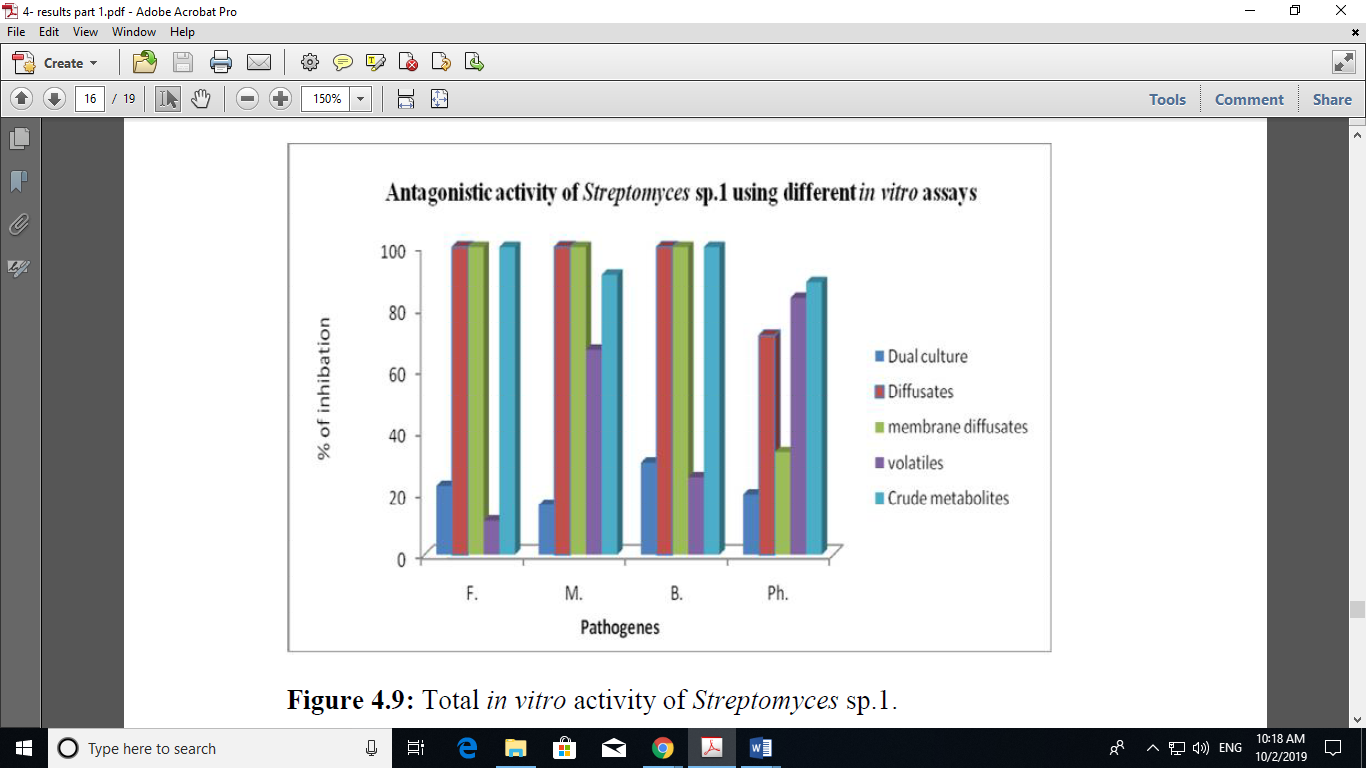


Figure 9: Total in vitro activity of *Streptomyces* sp.1.


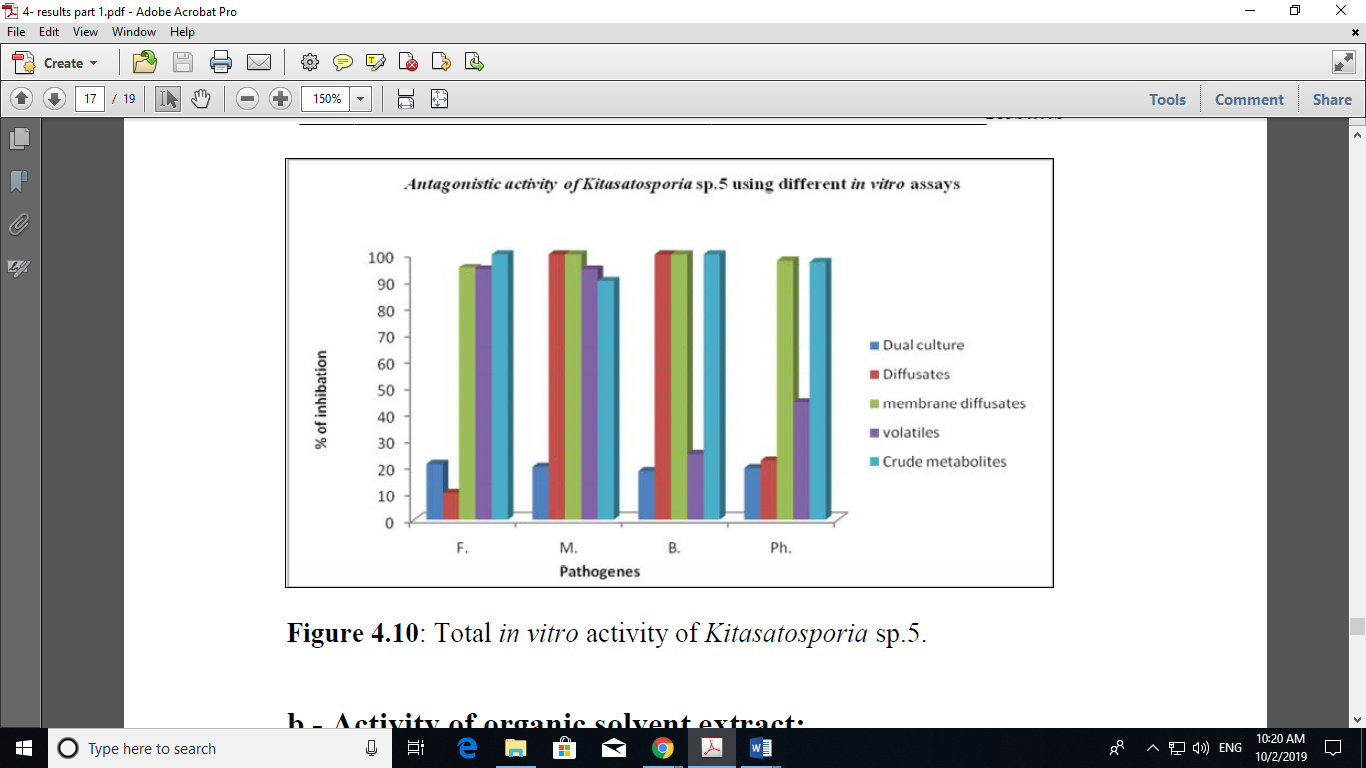


Figure 10: Total in vitro activity of *Kitasatosporia* sp.5.
